# Frequent cleft lip and palate in families with pathogenic germline *CDH1* variants

**DOI:** 10.3389/fgene.2022.1012025

**Published:** 2022-09-28

**Authors:** Benjamin L. Green, Grace-Ann Fasaye, Sarah G. Samaranayake, Anna Duemler, Lauren A. Gamble, Jeremy L. Davis

**Affiliations:** ^1^ Surgical Oncology Program, Center for Cancer Research, National Cancer Institute, National Institutes of Health, Bethesda, MD, United States; ^2^ Genetics Branch, Center for Cancer Research, National Cancer Institute, National Institutes of Health, Bethesda, MD, United States

**Keywords:** cleft lip, cleft palate, CDH1, E-cadherin, cleft lip/palate, hereditary diffuse gastric cancer syndrome

## Abstract

Pathogenic and likely pathogenic (P/LP) germline variants in the tumor suppressor gene *CDH1* (E-cadherin) result in increased lifetime risk of diffuse-type gastric cancer and lobular breast cancer. *CDH1* variants are also associated with hereditary cleft lip and palate (CLP), the mechanism of which is not well understood. We sought to determine the prevalence of CLP in families who carry P/LP *CDH1* variants. Patients with P/LP *CDH1* variants who were enrolled in a prospective clinical trial were reviewed (NCT03030404). The cohort included 299 individuals from 153 families that had 80 unique P/LP variants in *CDH1*. The rate of CLP was 19% (29/153) in families reporting CLP in at least one family member, and 2.7% (8/299) among individuals with confirmed germline *CDH1* P/LP variants. There were 22 unique variants in *CDH1* among the 29 families that reported CLP, or a CLP rate of 27.5% per variant (22/80). 10 of the variants were not previously reported to be associated with CLP. We observed that 24% (7/29) of CLP-associated gene variants involved large-scale (≥1 exon) deletions. Among families with CLP, 69% (20/29) had a member diagnosed with gastric cancer, and 79% (23/29) had a member with breast cancer, which were similar to rates observed in non-CLP families (*p* >0.3 for both). Our analysis suggests that the prevalence of CLP in families with germline *CDH1* P/LP variants was high in this large cohort, and there was no genotype-phenotype pattern. Genetic testing for *CDH1* variants should be considered in families with CLP and history of either diffuse-type gastric or lobular breast cancer.

## Report

E-cadherin is a glycoprotein involved in maintaining the integrity of mucosal epithelium via *trans*-homophilic binding at cell-cell junctions (Takeichi, 2014; Mendonsa et al., 2018). Germline pathogenic or likely pathogenic (P/LP) variants in the *CDH1* gene, which encodes E-cadherin, lead to the Diffuse Gastric and Lobular Breast Cancer (DGLBC, formerly hereditary diffuse gastric cancer, HDGC [MIM: 137215]) syndrome with an autosomal dominant pattern of inheritance. Lifetime disease penetrance estimates for gastric cancer and breast cancer in patients bearing a P/LP variant in *CDH1* are approximately 25–42% and 42–55%, respectively (Roberts et al., 2019; Xicola et al., 2019).

In addition to cancer phenotypes, *CDH1* variants are associated with Blepharocheilodontic syndrome (MIM: 119580) and cleft lip and palate (CLP). CLP, the most common congenital craniofacial abnormality, is a uni- or bilateral non-union of pharyngeal arch 1 structures and occurs in approximately 1 in 700 live births (Dixon et al., 2011). Most cases of CLP are idiopathic, but CLP may also present in the context of certain congenital syndromes (Venkatesh, 2009; Ghoumid et al., 2017). E-cadherin protein expression in the developing frontonasal prominence reportedly increases during weeks four–six of embryonic development (Frebourg et al., 2006), and epithelial cell adhesion is an important contributor to proper development of this structure (Cox et al., 2018). A prior study reported an association between variants in the linker regions of the E-cadherin protein and CLP, however, no mechanistic evidence has been provided to explain this phenomenon (Selvanathan et al., 2020).

To evaluate the association between *CDH1* variants and CLP, we analyzed a large single-institution cohort of 299 patients with confirmed *CDH1* P/LP variants enrolled in a prospective natural history study from 2017 through 2021. A total of 299 individual study participants were enrolled (211 female, 88 male) from 153 different families, the majority of whom identified as White (Table 1). Although the individual rate of CLP among patients with germline *CDH1* P/LP variants was 2.7% (8/299), 19% (29/153) of families identified at least one relative with CLP (Median: 1, range 1–5). Of the study participants and their relatives identified with CLP (*n* = 47), 15 were positive for a P/LP variant in *CDH1,* 1 was an obligate carrier, and 31 were untested but at-risk to carry the familial *CDH1* variant. Individuals with CLP were 45% (21/47) female, 19% (4/21) of whom had a personal history of breast cancer. Advanced gastric cancer was identified in 13% (6/47) of individuals with CLP. For families with CLP, 69% (20/29) reported at least one member with advanced gastric cancer, and 79% (23/29) reported breast cancer, which were similar to rates observed in non-CLP families (breast cancer *Χ* ^2^ = 0.33, *p* = 0.566; gastric cancer *Χ* ^2^ = 0.33, *p* = 0.567).

**TABLE 1:** Family demographic and variant characteristics of CLP and non-CLP cohorts.

Characteristic, n (%)	CLP N = 29	Non-CLP N = 124
Family history of breast cancer		
Yes	23 (79)	92 (74)
No	6 (21)	32 (26)
Family history of gastric cancer		
Yes	20 (69)	92 (74)
No	9 (31)	32 (26)
Race		
White	28 (97)	111 (90)
Black	—	3 (2)
Asian	—	3 (2)
Hispanic	—	1 (1)
Multiple/Other	1 (3)	6 (5)
Variant domain		
All	2 (7)	3 (2)
Pre	—	7 (6)
Pro	2 (7)	10 (8)
Cadherin 1	7^a^ (24)	18^a^ (15)
Cadherin 2	—	8 (6)
Cadherin 3	1 (3)	10 (8)
Cadherin 4	6 (21)	23^b^ (19)
Cadherin 5	2 (7)	16^c^ (13)
Transmembrane	4 (14)	20 (16)
Cytoplasmic	5 (17)	9 (7)
Variant Type		
Deletion	8 (28)	9 (7)
Frameshift	4 (14)	33 (27)
Missense (Cryptic Splice)	1 (3)	21 (17)
Nonsense	7 (24)	40 (32)
Splice site (Canonical)	9 (31)	21 (17)

a Two variants are in the Pro-EC1, linker region.

b One variant is in the EC3-EC4 linker region.

c One variant is in the EC4-EC5 linker region.

**FIGURE 1:**
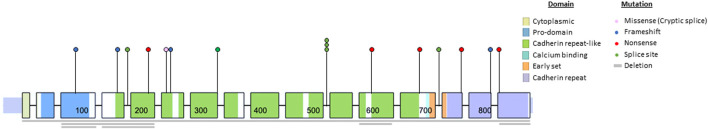
Map of variants in *CDH1* for patients reporting family history of CLP.

Next, we analyzed the *CDH1* genotype of the cohort (Figure 1). There were 80 unique *CDH1* P/LP variants among 153 different families. Of the 29 families that reported CLP, there were 22 unique variants in *CDH1*, 10 of which had not been associated previously with CLP (Table 2). The rate of CLP per unique *CDH1* P/LP variant was 27.5% (22/80). Truncation of E-cadherin was predicted in 55% (16/29) of families reporting CLP based on either nonsense or frameshift variants in *CDH1* (Table 2). An additional 24% (7/29) of CLP families had large deletions of ≥1 exon, including two families that were heterozygous for complete *CDH1* gene deletion. Interestingly, there were two other families in the CLP-negative cohort heterozygous for the same complete *CDH1* gene deletions that denied a known history of CLP. In contrast, there was only 1 missense cryptic splice variant in CLP-positive families (3%) compared with 21 missense mutations in the CLP-negative families (17%). The missense cryptic splice variant in the CLP-positive subgroup was not located within a cadherin-repeat linker region. Surprisingly, variants located at EC-EC linker regions were found in families without a history of CLP. The most common location for a *CDH1* variant in both subgroups was in EC4. The frequency of variants of intracytoplasmic or transmembrane domains were similar in both CLP-positive and CLP-negative groups.

**TABLE 2:** *CDH1* variant genotype for each family with CLP.

Family	*CDH1* variant	Variant domain	Variant type	Amino acid change	Prior report in CLP
1	5′UTR_3′UTRdel	All	Deletion (Complete)	—	None
2	5′UTR_3′UTRdel	All	Deletion (Complete)	—	None
3	c.261del	Cadherin pro	Frameshift	Arg87fs	None
4	Deletion (Exon 3)	Cadherin pro	Deletion (Large)	—	None
5	Deletion (Exons 3–5)	Cadherin pro through extracellular Cadherin 1	Deletion	—	None
6	Deletion (Exons 4–5)	Cadherin pro through extracellular cadherin 1	Deletion	—	None
7	Deletion (Exon 16)	Cytoplasmic	Deletion (Large)	—	None
8	EX16_3′UTRdel	Cytoplasmic	Deletion (Large)	—	None
9	c.2430del	Cytoplasmic	Frameshift	Phe810fs	Present
10	c.2474dup	Cytoplasmic	Nonsense	p.Pro826fs	Present
11	c.2287G>T	Cytoplasmic	Nonsense	Glu763Ter	Present
12	c.480_486del	Extracellular cadherin 1	Frameshift	p.Ile161AlafsTer52	None
13	c.640del	Extracellular cadherin 1	Nonsense	Leu214Ter	None
14	c.532-1G>C	Extracellular cadherin 1	Canonical splice	—	Present
15	c.720del	Extracellular cadherin 1	Frameshift	Asn240fs	None
16	c.715G>A	Extracellular cadherin 1	Missense (Cryptic splice)	Gly239Arg	Present
17	c.1137G>A	Extracellular cadherin 3	*Canonical splice	—	Present
18	c.1565+2dupT	Extracellular cadherin 4	Canonical splice	—	Present
19	c.1565 + 1G>C	Extracellular cadherin 4	Canonical splice	—	Present
20	c.1565 + 1G>A	Extracellular cadherin 4	Canonical splice	—	Present
21	c.1565 + 1G>A	Extracellular cadherin 4	Canonical sSplice	—	Present
22	c.1565 + 1G>C	Extracellular cadherin 4	Canonical splice	—	Present
23	c.1565 + 1G>A	Extracellular cadherin 4	Canonical splice	—	Present
24	Deletion (Exon12)	Extracellular cadherin 5	Deletion (Large)	—	None
25	c.1792C>T	Extracellular cadherin 5	Nonsense	Arg598Ter	Present
26	c.2064_2065del	Transmembrane	Nonsense	p.Cys688Terfs	Present
27	c.2064_2065del	Transmembrane	Nonsense	p.Cys688Terfs	Present
28	c.2064_2065del	Transmembrane	Nonsense	p.Cys688Terfs	Present
29	c.2165-1G>C	Transmembrane	Canonical splice	—	Present

*a synonymous last nucleotide variant that abolishes the donor splice site.

Here, we have reported the largest known single-institution analysis of CLP prevalence in subjects with germline *CDH1* P/LP variants. The rarity of DGLBC syndrome and *CDH1* P/LP variants presents challenges for any analysis. A prior study of *CDH1* variant data pooled from the literature and public genetic variation databases found that 13% of *CDH1* variants were associated with syndromic CLP (only DGLBC and Blepharocheilodontic syndrome) and non-syndromic CLP (Selvanathan et al., 2020). Our dataset, in contrast, allowed for CLP status to be systematically collected. We were able to determine that 27.5% of unique *CDH1* P/LP variants were associated with CLP. Additionally, 19% of families with germline *CDH1* P/LP variants reported at least one relative with CLP. These data demonstrate that CLP may be more prevalent in families with *CDH1* P/LP variants than previously described.

Identification of individuals with a *CDH1* P/LP variant provides opportunities for cancer risk reduction and early detection. Due to the high incidence of CLP in the general population, a diagnosis of isolated CLP at birth would be insufficient to recommend germline *CDH1* genetic testing. Detailed individual and family criteria for *CDH1* germline genetic testing have been developed by the International Gastric Cancer Linkage Consortium (Blair et al., 2020). Of the nine specific testing criteria, only one addresses CLP which recommends *CDH1* testing for individuals with diffuse gastric cancer at any age and a personal or family history of CLP. Based on this report, it appears quite reasonable to expand the criteria to include a recommendation for *CDH1* genetic testing in individuals with lobular breast cancer at any age with a personal or family history of CLP. Another consideration is that in families with features of hereditary cancer, there will be relatives with syndrome associated cancers who are deceased or uninterested/unable to undergo genetic testing. Therefore, we suggest that *CDH1* genetic testing criteria also include testing for unaffected individuals with a family history of CLP and diffuse gastric cancer or lobular breast cancer.

Genotype-phenotype correlations have been elusive for *CDH1*. We found no difference in the rates of CLP in families reporting a history of gastric or breast cancer. Functionally, E-cadherin can form hetero- and homodimers on the cell surface and initiates intracellular signal transduction via β-catenin signaling and cytoskeletal modulation (Mendonsa et al., 2018). A previous study suggested mechanistic associations that might explain phenotypic differences between CLP and cancer development, specifically implicating linker regions of E-cadherin enriched for CLP-associated variants (Selvanathan et al., 2020). However, we found no evidence of region-specific variants that correlated with the presence of CLP. The CLP-positive subgroup demonstrated variants throughout the entire gene, including two patients with full *CDH1* gene deletions which had not been reported previously. Interestingly, there were two additional families with full *CDH1* gene deletions that reported no CLP. In addition, the only missense mutation in the CLP + group was a known cryptic splice site, generating premature termination codon that potentially resulted in reduced abundance of *CDH1* mRNA via the nonsense-mediated decay (NMD) pathway (Kaurah et al., 2007; Karam et al., 2008). Together, these findings suggest that quantity, not quality, of functional E-cadherin may be a driver of CLP phenotype in *CDH1* P/LP carriers, and that CLP is likely a multifactorial phenotype.

## Materials and methods

The study was conducted in accordance with the Declaration of Helsinki (as revised in 2013). Patients were enrolled in National Institutes of Health (NIH) protocol number 17-C-0043 (NCT ID: NCT03030404) from 2017 to 2021. The study was approved by the institutional review board of the National Institutes of Health (reference number 385481) and informed consent was taken from all patients. Patients were enrolled if they had positive genotyping for a P/LP variant in *CDH1*. Patients had genetic testing at a CLIA certified lab. Results were reviewed by a certified genetic counselor. All data were analyzed by SPSS version 25^®^ (IBM, IL, United States). Chi-squared statistical test was used where appropriate.

## Summary

Approximately 1 in 5 families with germline *CDH1* pathogenic variants identified a family member with cleft lip/palate. This rate of cleft lip/palate associated with germline *CDH1* variants should be incorporated into considerations for genetic testing in patients with a personal or family history of diffuse gastric cancer or lobular breast cancer.

## Data Availability

The original contributions presented in the study are included in the article/supplementary materials, further inquiries can be directed to the corresponding author.
